# Using Latent Profile Analysis to Evaluate Preferences for Chronic Wasting Disease Management Options among Different Hunter Types

**DOI:** 10.3390/ani12202751

**Published:** 2022-10-13

**Authors:** Elena C. Rubino, Christopher Serenari

**Affiliations:** 1 College of Forestry, Agriculture and Natural Resources, University of Arkansas at Monticello, Monticello, AR 71656, USA; 2 Arkansas Forest Resources Center, Division of Agriculture, University of Arkansas System, Monticello, AR 71656, USA; 3 Department of Biology, Texas State University, San Marcos, TX 78666, USA

**Keywords:** CWD, deer management, discrete choice experiment, hunter typology

## Abstract

**Simple Summary:**

Extensive research has explored hunters’ support for chronic wasting disease management, but many studies do not account for differences between types of hunters nor the tradeoffs hunters make in their decision-making about management alternatives. To address this, we used data from a survey of Texas hunters to create categories of hunters and explore their preferences for chronic wasting disease management based on these categories. Across five hunter categories, most hunters support disease management, although their attitudes towards Texas Parks and Wildlife Department varied. Different hunter categories had different preferences for chronic wasting disease management policies. Wildlife agencies can refer to our study findings to create preferred chronic wasting disease management policies and better communicate about them.

**Abstract:**

Wildlife agencies seek to understand how hunters have and will respond to chronic wasting disease (CWD) management policies because of the vital role hunters play in deer management efforts. As such, dozens of studies have examined the human dimensions of CWD management and policy to assess stakeholder support for management alternatives and reveal what drives support. However, most of these studies have not (1) fully explored the heterogeneity that exists among hunters, and (2) accounted for the tradeoffs that agencies and hunters must make with regard to deer management and potential CWD policy alternatives. To address these deficiencies, we used latent profile analysis to create different typologies of hunters based on a survey of Texas hunters, then analyzed discrete choice experiments investigating the CWD management preferences of these typologies. Across five hunter typologies, we found strong overall support for CWD management, although attitudes towards Texas Parks and Wildlife Department were variable. Preferences for CWD management policies greatly differed between each hunter typology. Wildlife agencies can refer to our findings to better develop hunter-preferred CWD management policies and identify areas of compromise between typologies. Our results also provide agencies with insights regarding how to better communicate about CWD management with different types of hunters.

## 1. Introduction

Wildlife agencies in North America continue to be concerned about the spread and management of chronic wasting disease (CWD), a fatal neurological disease affecting cervid species [1]. In addition to concerns related to challenges associated with managing the disease ecology of CWD, these agencies are also faced with managing human responses to CWD and CWD management [2]. Handling stakeholder responses to CWD management involves addressing multiple dimensions, including the public’s concerns about CWD-related risks to humans and deer populations, the public’s trust in the wildlife agency, and the public’s attitudes towards current and future CWD management [2,3,4,5,6]. Understanding how hunters have and will respond to CWD management policies is particularly crucial to agencies because of the vital role hunters play in deer management. Hunters often directly assist in CWD management efforts such as general herd depopulation, spatially targeted harvesting in CWD-affected areas, and age and/or sex-specific targeted harvesting [6,7].

Understanding stakeholder support for CWD management alternatives and what drives that support is crucial to CWD management and policy. Evidence suggests that hunter beliefs about the likelihood of deer herd reduction strongly influence support for the management strategy, indicating that hunters must believe in the efficacy of management alternatives to support them [3]. Furthermore, institutional trust, trust in agency information/management, and support for/perceived efficacy of CWD regulations can influence hunters’ attitudes about CWD management [6]. For example, shared values (or a lack thereof) and agency trust in information and technical competence have served as drivers of perceptions of agency management [6]. Risk perceptions related to CWD also play a pivotal role in these interactions [2,3,4,8]. Specifically, multiple studies have demonstrated the strong relationships between perceived health risks, agency trust, and hunter acceptance of CWD management actions [9]. In these cases, higher CWD risk perceptions were often linked to lower trust in the wildlife agency, which influenced the management actions that were supported by hunters.

As indicated above, social science research on deer management often consists of analyses that document factors influencing attitudes towards and preferences for CWD management, e.g., [2,3,8]. However, two core deficiencies arise from these common approaches that prevent wildlife agencies from realistically addressing the preferences of and efficiently communicating with different types of hunter stakeholders, which are vital to hunter satisfaction and support. First, most studies do not fully explore the heterogeneity that often exists among hunters. Instead, they tend to analyze hunter respondents as a homogenous group and, by default, results of such studies provide us with relationships based on averages of this whole group. For example, Harper et al. [2] determined that support for sharpshooting was greater among hunters who trusted the Illinois Department of Natural Resources and perceived higher CWD risks to deer and human health. Similarly, Meeks et al. [8] found that levels of hunter acceptability of alternative CWD management actions were influenced by deer and human health concerns, regulatory concerns, trust in the wildlife agency, and experience hunting out of state. These results paint a broad picture of relationships for a typical respondent, but they fall short of identifying patterns within the diversity of hunters and their heterogenous behaviors, attitudes, and preferences. Identifying hunter typologies can provide wildlife agencies with an understanding of the extent of this diversity and how variations in attitudes and preferences interact [10]. Thus, creating typologies of hunters based on, for example, their motivations for hunting, e.g., [10] or attitudes towards and support for management, e.g., [11], can inform wildlife agencies on how they can better satisfy and effectively communicate with different types of hunters.

A second core issue in common approaches to exploring support for CWD management is that few studies account for the tradeoffs that state agencies and hunters routinely need to make with regard to deer management and potential policy alternatives [8,12]. Although some recent deer management research has employed methods to elicit tradeoffs between social and biological goals [13,14], most surveys rely on Likert scales, which reveal attitudes towards distinct policy attributes in a straightforward manner for both administration and analysis. However, it is argued that Likert scales have little theoretical basis and are known for their inability to capture tradeoffs [15]. Methods from decision-making research that are underpinned by random utility theory (i.e., the well-tested theory of choice behavior) better model actual decision making, and using these methods can result in more accurate estimates of overall preferences, measures of strength of preference, and accounting for realistic tradeoffs in constraints between alternatives [15].

Deficiencies in research on support for CWD management led us to ask the following research question: what CWD management alternatives maximize satisfaction among different types of hunters? To answer this question, we sought to develop models of decision-making related to CWD management alternative preferences for multiple, heterogeneous types of hunters. We used a latent profile analysis to first segment Texas hunters into typology profiles based on variables known to influence perceptions of CWD management, then we analyzed each profile’s responses to a discrete choice experiment to explore heterogeneity in choice behavior among hunter profiles. This dual approach is novel in that it addresses the two core issues in most human dimensions of CWD management research, thus generating findings that can be used to inform wildlife agency policy development that appeals to specific hunter segments or compromise among segments, as well as providing agencies with information needed to generate communications to targeted hunter segments.

### 1.1. Latent Profile Analysis

Latent profile analysis (LPA) is used to identify naturally occurring, unobserved classifications of respondents (i.e., latent profile) based on continuous, observed variables (e.g., responses to survey items). This method creates data-derived classifications and, unlike other classification techniques (e.g., cluster analysis), the analysis allows for mathematical evaluation of proposed LPA models [16]. Key parameters in the statistical expression (Equation (1)) include *μ_ik_* and *σ_ik_* as *k* profile-specific means and variances for variable *i*. Profile density, or the proportion of N respondents belonging to profile *k* is represented as *π_k_*. Latent profile analyses assume that samples are drawn from a heterogeneous population and produce data that are a combination of *K* profile-specific distributions [17].

(1) $$\sigma_{i}^{2}=\overset{K}{\underset{k=1}{\sum }}\;\;\pi_{k}\left(\mu_{ik}-\mu_{i}\right){\;}^{2}+\overset{K}{\underset{k=1}{\sum }}\;\;\pi_{k}\sigma_{ik}^{2}$$

### 1.2. Utility Theory and Discrete Choice Experiments

Discrete choice experiments are grounded in utility theory, where we can model the utility (i.e., satisfaction, *U*) that a decision-maker (*n*) (i.e., a respondent) obtains from choosing a specific utility-maximizing alternative (*j*) from a total set of *J* alternatives (Equation (2)). Within this model, for all *j* alternatives (∀*j*), utility is comprised of *V_nj_*, which is known by the researcher up to some parameters, and *ε_nj_*, which are parts that are unknown by the researcher and treated as random and distributed independently, identically extreme value [18].

(2) $$U_{nj}=V_{nj}+\varepsilon_{nj}\;\forall j$$

In our application, this method reveals the decision-making behind how respondents trade-off between potential CWD management policies by asking respondents to select which policy they preferred between two potential policies.

## 2. Methods

### 2.1. Study Area

In 2012, the first case of CWD in Texas was identified in free-ranging mule deer (*Odocoileus hemionus*) in western Texas. The disease was then detected in white-tailed deer (*O. virginianus*) for the first time in Texas in a Medina county breeding facility in 2015 [19]. At the time of the study, CWD was present in eight counties (Dallam, El Paso, Hartley, Hudspeth, Kimble, Medina, Uvalde, and Val Verde Counties) spanning five zones throughout the state. As of July 2022, CWD has spread to 14 counties and been found in captive and free-range cervids including white-tailed deer, mule deer, red deer (*Cervus elaphus*), and elk (*C. canadensis*) [20].

### 2.2. Survey Design and Implementation

We developed a survey instrument to administer to hunters throughout the state of Texas who hunt white-tailed deer, mule deer, red deer, sika deer (*C. nippon*), and/or elk. We cognitively pretested the survey according to Alaimo et al. [21], and the survey was approved by the Texas State University Institutional Review Board (#7222). Screening questions at the beginning of the survey confirmed that respondents lived in Texas, had purchased a Texas hunting license within the past five years, and had hunted at least one of the aforementioned species in Texas within the past five years. The survey contained questions regarding current hunting and CWD testing behaviors, CWD-related risk perceptions [22], and attitudes towards Texas Parks and Wildlife Department’s (TPWD) approach to CWD management [5,8,23] and CWD management policies [24], as well as respondents’ preferences for CWD-related communication [25] and demographic information. All questions included an “I don’t know” response, which was then excluded from analyses. 

For the discrete choice experiment, each “policy” consisted of attributes including a population reduction target in CWD Zones, carcass movement restrictions, mandatory CWD testing of hunter-harvested deer strategies, and a potential ban on releasing captive deer into free-ranging populations. Realistic potential policy attributes and associated levels (Table 1) were developed in conjunction with TPWD biologists. Given the four attributes and 10 associated levels, we used SAS statistical software to generate an optimal design to maximize the information derived from the choice experiment while limiting cognitive fatigue (D-efficiency = 98.17) [26], which resulted in two versions of the choice experiment where each respondent answered three choice questions.

Texas Parks and Wildlife Department provided us with a sampling frame of current Texas hunting license holders which we used to generate a simple random sample [27] for survey dissemination. Given the population of Texas hunters statewide, we required approximately 379 valid responses to make robust statistical inferences at a 95% confidence level. Beginning on 12 September 2020, we mailed 9492 sampled hunters a survey packet containing an introductory letter (with a URL to take the survey online), a paper version of the survey, and a postage-paid return envelope. Each potential respondent also received up to two follow-up postcard reminders. We ceased data collection on 20 January 2021, and then mailed a brief demographic survey to 500 nonrespondents in order to assess potential nonresponse bias.

### 2.3. Data Analysis

We sought to investigate patterns of memberships in latent profile groups based on variables that have been found to influence perceptions of CWD management, namely CWD-related risk perceptions and attitudes towards and trust in wildlife agencies and their CWD regulations [2,3,8]. We used principal factor analysis to reduce multiple variables into smaller sets of underlying factors [28]. Following Meigs [28], we used an eigenvalue threshold of 1.0 to keep factors. We then used factor loadings to produce weighted scores measuring respondents’ beliefs about TPWD’s inclusion of stakeholders and TPWD’s competence regarding CWD management (Table 2). This process resulted in four continuous variables used in our model. We used Stata BE 17′s -*gsem*- command to conduct the LPA. We determined the best-fit LPA model based on Akaike’s information criterion and Bayesian information criterion and identified latent profiles of respondents, including the proportion of respondents assigned to each profile [29]. Then, we used a vector (-*predclass*-) for predicting profile memberships, which assigned each respondent to a latent profile [30].

We utilized these predicted profile memberships to analyze preferences among different profiles for CWD management policies. For this analysis of the discrete choice experiment, we coded the selected policy (i.e., the dependent choice variable) as a “1” if the policy was selected and “0” if not selected. We effects-coded the categorical choice experiment levels in order to estimate each attribute level while avoiding multi-collinearity among dummy variables (Table 1). As such, one level of an attribute is embedded as −1 and its coefficient is calculated as the negative sum of the coefficients of other attribute levels [26]. We analyzed the data using a fixed-parameter logit model using the -*logit*- command in Stata BE 17 [26].

## 3. Results

Our data collection effort yielded 503 completed responses, equating to a response rate of 5.3%. This number of responses allowed us to make robust statistical inferences at the 95% confidence level. Respondents were predominantly male (*n* = 451; 91.48%) and their ages ranged from 18 to 87 ( $\bar{x}$ = 55.03, SD = 16.85). Respondents mostly resided in rural settings (55.28%, *n* = 272), and 31.30% (*n* = 154) and 13.41% (*n* = 66) resided in suburban and urban settings, respectively. Most respondents 62.55% (*n* = 309) had completed some college or held a bachelor’s degree, whereas 19.64% (*n* = 97) held a graduate degree, and 17.81% (*n* = 88) had a high school degree or less formal schooling. We received 33 nonresponse bias survey responses and there were no significant differences between respondents and nonrespondents with regard to age, residential setting, or levels of formal education. There was, however, a smaller proportion of female hunters among our respondents (X^2^ (1, *n* = 493) = 6.80, *p* = 0.01).

We tested multiple models to evaluate the model fit for different class solutions generated by the latent profile analysis. The five-class solution with predictor variables yielded the most parsimonious model according to both the Akaike’s information criterion and Bayesian information criterion (Table 3). Although none of the predictor variables (age, gender, residential setting, hunting location, and a hunter behavior change measure) were significantly different between profiles, their inclusion improved the model fit. Profiles 1 and 2 each represented slightly greater than one-third of our respondents. Respondents classified into Profile 1 were moderately-to-very concerned about free-range deer contracting CWD in Texas, strongly agreed that CWD regulations are necessary, and generally agreed that TPWD involves hunters in CWD management and that TPWD competently addresses CWD management. Profile 2 respondents were also moderately-to-very concerned about CWD and strongly agreed that CWD regulations are necessary; however, they did not generally agree that TPWD’s CWD management involves hunter stakeholders, nor is it competently addressed. Profile 3 represented approximately 17% of respondents and this profile corresponds with respondents who are slightly-to-moderately concerned about CWD, agree that regulations are necessary, but again do not generally agree that TPWD involves hunters in nor competently addresses CWD management. Profiles 4 and 5 combined represent less than 8% of respondents. Respondents classified into Profile 4 are only slightly concerned about CWD and they disagree with CWD regulations and TPWD’s approach to CWD management. Profile 5 respondents are slightly-to-moderately concerned about CWD, but fairly neutral about the necessity of regulation and TPWD’s approach to CWD management.

For the logit models (one for each of the five latent class profiles), positive coefficients represented an increased probability that respondents would select a management policy and negative coefficients represented a decreased probability of management policy selection. The effects-coding in the models provides parameter estimates for each choice experiment attribute. In the Profile 1 model, the probability that a management policy was selected decreased if the policy included population reductions of 60% in the CWD management zone or a ban on the release of captive deer into free-ranging deer populations (Table 4). Profile 2 respondents were less likely to select a management policy if it included statewide mandatory CWD surveillance of hunter-harvested deer for 1 or 2 weekends per season. The probability that Profile 3 respondents selected a management policy decreased if the policy included population reductions of 40% in the CWD management zone, but increased if the policy included a ban on release of captive deer into free-ranging deer populations. None of the management policy attributes were significant determinants of policy selection for Profile 4 respondents. The Profile 5 model indicated that respondents were less likely to select a management policy if it included statewide mandatory CWD surveillance of hunter-harvested deer for 1 weekend per season and were more likely to select a management policy if it included a ban on release of captive deer into free-ranging deer populations (Table 4).

## 4. Discussion

Our results indicate a substantial proportion of hunters (across multiple typologies) generally support CWD management. The majority of hunters (greater than 75%) fell into Profiles 1 and 2, meaning they were all moderately to very concerned about CWD and strongly agreed that CWD regulations are necessary, although their attitudes towards TPWD differed. Additionally, respondents within Profiles 3 and 5 were still slightly to moderately concerned about CWD and agreed or were neutral towards regulations. Like many hunters in Tennessee [8], New York [31], and Minnesota [6], Texas hunters appear to be well-positioned to accept CWD management policies. This finding is promising for TWPD in that it suggests the vast majority of hunters recognize the importance of CWD management and will likely support TPWD’s CWD management efforts, even when attitudes towards the agency are not resoundingly positive. This is critical because agencies often rely on hunters for some CWD management, including keeping deer populations at target levels and limiting the spread of CWD via appropriate deer carcass disposal and limiting carcass transportation across county lines [8].

Hunters within profiles 2, 3, and 5, who tend to agree with CWD management but are less positive towards TPWD’s approach, highlight a potential opportunity for TPWD to build trust and support among hunters. This may be achieved by sharing communications that emphasize how TPWD’s policies are resulting (or will result) in progress towards containing CWD, generating greater confidence in TPWD [3]. Given the concern about CWD across these profiles (which is also consistent with studies in other states [5,32]), communications should focus on the implications of CWD on deer populations and deer health, thus providing hunters with a better understanding of how CWD may impact deer and what it may mean for hunters [33]. These profiles are segments that would be ideal for such targeted communications because they would likely be easier to shift to a more supportive/positive stance than Profile 4 respondents, who clearly oppose CWD regulations and TPWD’s efforts [34]. Respondents within Profile 4 represent the minority of respondents (<5%), and it would likely be very difficult to shift this group to a more supportive stance given their distrust of TPWD [2,6]. As such, it may not be worth attempting given the time and resources necessary.

We found that our respondent profiles are helpful in understanding hunter preferences for management because they illuminate the array of policy preferences among different hunter groups [11], including some areas of shared support and potential opposition. For example, respondents in Profiles 2 and 3 are similar in that neither group agrees that TPWD involves hunters in, nor competently addresses CWD management and their preferences for management reflect this distrust in the agency. Profile 2 respondents do not want statewide mandatory CWD surveillance of hunter-harvested deer for 1 or 2 weekends per season (Profile 5 appears to only not prefer statewide mandatory CWD surveillance of hunter-harvested deer for 1 weekend per season, likely due to the small segment representation). Profile 3 respondents are supportive of a ban on release of captive deer into free-ranging deer populations (which is an action that does not require active TPWD management of deer). It should not be a surprise that Profile 4 does not prefer any management alternatives. 

Although each hunter profile has a unique set of management alternatives that maximizes utility, and there is little overlap between profiles, agencies can use such results to find areas of compromise between core hunter typologies (in this case, likely Profiles 1–3 due to the size of these groups) [35]. This potential for compromise is particularly relevant given that our findings mostly feature the management alternatives that are not supported among certain groups. This also highlights the benefit of our discrete choice method that accounts for tradeoffs [15]. Compromise might look like avoiding policies (e.g., avoiding policy attributes entirely, or avoiding more polarizing levels of attributes) that are strongly opposed, given tradeoffs in other areas where support is more neutral. In fact, the tactic of framing policies as strategic acts of compromise among groups has proven effective in generating support for legislation. Rademacher [36] concluded communicating acts of strategic compromise at different points in the legislative process successfully generated support among legislatures and voters for multiple major tax reforms in the United States. A similar strategy could be employed by wildlife agencies to shore up support for comprehensive CWD management policies that are of interest to agencies. For example, the probability that a management policy was selected decreased if the policy included population reductions of 60% in the CWD management zone for Profile 1 and 40% in the CWD management zone for Profile 3. Therefore, perhaps 20% reductions in the CWD management zone are a reasonable option for agencies. Additionally, statewide carcass movement restrictions were not significantly preferred or not preferred among any hunter profiles, indicating that they may be a neutral choice for agencies to pursue and may not yield policy conflict. By framing their discourse to emphasize the compromises being made among the agency and hunter segments, TPWD may be able to gain broader support across hunter typologies.

Our findings related to hunter preferences for management alternatives underscore the need for wildlife agencies to weigh hunter opposition to a management alternative against the management alternative’s potential impact on CWD. For example, although Profile 3 respondents do not prefer population reductions of 40% in the CWD management zone, TPWD will have to eventually determine if population reductions to that extent are necessary to control CWD in an area. Similarly, a ban on the release of captive deer into free-ranging deer populations is supported by respondents in Profiles 3 and 5, but opposed by Profile 1 respondents. A potential ban on the release of captive deer is meant to address the fact that chronic wasting disease incubation can be several years. Thus, asymptomatic CWD-positive deer from a captive facility can unknowingly be transported across multiple facilities or released into a native deer population [37]. Given these risks associated with captive deer facilities, TPWD will need to evaluate stakeholder support for and opposition to a ban against the effects that the ban may have on CWD’s spread across the state. These considerations also highlight how communications can be used to influence support for a biologically effective but socially less-preferred management alternative [38]. Specifically, results from this study can be used to strategically target communications about the efficacy of given management alternatives towards certain profiles of hunters.

## 5. Conclusions

Hunters are a heterogenous group in terms of their behaviors and attitudes (Andersen et al., 2014), as well as their preferences for CWD management strategies. Wildlife agencies can refer to our findings to inform development of hunter-preferred CWD management policies and identify areas of compromise between hunter segments. Additionally, our results provide agencies with insights regarding how to better market their management policies towards specific skeptical hunter segments, thus increasing overall support for CWD management.

## Figures and Tables

**Table 1:** Attributes and levels of the choice experiment and effects coding used in logit model analysis of Texas hunter CWD management preferences.

Attribute/Level	Effects Coding	
**Population reduction**	PopReduction40	PopReduction60
20% population reductions in CWD Zones	−1	−1
40% population reductions in CWD Zones	1	0
60% population reductions in CWD Zones	0	1
**Carcass movement restrictions**		
Statewide	1	
CWD Zones Only	−1	
**CWD testing**	Testing1Week	Testing2Week
Mandatory CWD testing of hunter-harvested deer only in CWD Zones	−1	−1
Statewide mandatory CWD surveillance of hunter-harvested deer for 1 weekend per season	1	0
Statewide mandatory CWD surveillance of hunter-harvested deer for 2 weekends per season	0	1
**Ban on release of captive deer into free-range deer populations?**		
Ban	1	
No ban	−1	

**Table 2:** Description of variables included in the latent profile analysis of Texas hunters.

Variable	Description	Coding
CWD concern	Concern about free-range deer contracting CWD in Texas	Higher values reflect greater levels of concern (scale 1–4)
Regulations necessary	Belief that TPWD’s regulations are necessary to protect deer populations in Texas from CWD	Higher values reflect greater levels of agreement that TPWD’s regulations are necessary (scale 1–5)
Hunter involvement	Belief that TPWD involves hunter stakeholders in CWD management	Score generated using principal factor analysis; higher values reflect greater levels of agreement that TPWD has clearly defined hunter responsibilities and expectations in CWD matters, is transparent with and inclusive of hunter stakeholders, and manages CWD in a way that is compatible with hunters’ personal visions of how CWD should be managed
Competent CWD management	Belief that TPWD competently addresses CWD management	Score generated using principal factor analysis; higher values reflect greater levels of agreement that TPWD uses the best available science and has the health and safety of wildlife in mind in CWD decision-making, and TPWD has adequately delivered CWD technical assistance that has been effective in preventing the spread of CWD.

**Table 3:** Results of the latent profile analysis of Texas hunters. For each profile, this table reports the coefficient (standard error) [95% confidence interval] for each variable used in the latent profile analysis.

	Profile 1 (*n* = 196; 38.97%)	Profile 2 (*n* = 186; 36.98%)	Profile 3 (*n* = 84; 16.70%)	Profile 4 (*n* = 22; 4.37%)	Profile 5 (*n* = 15; 2.98%)
CWD concern	3.33 ** (0.08) [3.18, 3.50]	3.27 ** (0.10) [3.07, 3.46]	2.87 ** (0.10) [2.69, 3.06]	1.97 ** (0.19) [1.60, 2.33]	2.61 ** (0.22) [2.17, 3.05]
Regulations necessary	2.00 ** (0.01) [1.98, 2.02]	2.00 ** (0.01) [1.98, 2.02]	1.00 ** (0.01) [0.97, 1.03]	−1.59 ** (0.02) [−1.64, −1.54]	0.00 (0.01) [0.97, 1.03]
Hunter involvement	0.84 ** (0.08) [0.68, 1.01]	−0.72 ** (0.11) [−0.91, −0.51]	−0.45 ** (0.10) [−0.64, −0.26]	−1.39 ** (0.17) [−1.72, −1.05]	−0.50 * (0.21) [−0.91, −0.10]
Competent CWD management	0.84 ** (0.09) [0.67, 1.01]	−0.57 ** (0.10) [−0.76, −0.38]	−0.32 ** (0.10) [−0.52, −0.12]	−1.75 ** (0.16) [−2.07, −1.43]	−0.53 * (0.23) [−0.98, −0.08]

Asterisks denote significance: (**) at the 1% level and (*) at the 5% level.

**Table 4:** Logit model results of Texas hunter CWD management preferences by latent profile classification.

Management Alternative	Coefficient (Standard Error)				
	Profile 1	Profile 2	Profile 3	Profile 4	Profile 5
Population reduction of 20% in CWD Zones ^a^	0.43	0.24	0.35	0.56	0.12
Population reduction of 40% in CWD Zones	0.50 (0.36)	0.02 (0.14)	−0.20 (0.10) *	0.04 (0.43)	−0.07 (0.10)
Population reduction of 60% in CWD Zones	−0.93 (0.32) **	−0.26 (0.14)	−0.15 (0.10)	−0.60 (0.41)	−0.05 (0.11)
Carcass movement restrictions in CWD Zones only ^a^	0.42	0.19	0.02	−0.26	−0.01
Statewide carcass movement restrictions	−0.42 (0.13)	−0.19 (0.10)	−0.02 (0.07)	0.26 (0.28)	0.01 (0.07)
Mandatory CWD testing of hunter-harvested deer only in CWD Zones ^a^	−0.98	0.67	0.38	−0.46	0.51
Statewide mandatory CWD surveillance of hunter-harvested deer for 1 weekend per season	−0.62 (0.34)	−0.37 (0.14) **	−0.19 (0.10)	0.47 (0.45)	−0.43 (0.10) **
Statewide mandatory CWD surveillance of hunter-harvested deer for 2 weekends per season	−0.36 (0.33)	−0.30 (0.15) *	−0.19 (0.10)	−0.01 (0.40)	−0.08 (0.10)
No ban on release of captive deer into free-ranging deer populations ^a^	0.97	0.07	−0.47	−0.39	−0.39
Ban on release of captive deer into free-ranging deer populations	−0.97 (0.24) **	−0.07 (0.10)	0.47 (0.07) **	0.39 (0.28)	0.39 (0.07) **
Constant	0.08 (0.23)	−0.02 (0.10)	−0.00 (0.07)	0.01 (0.28)	−0.01 (0.07)
Log likelihood	−60.19	−290.86	−616.86	−36.66	−554.29

Asterisks denote significance: (**) at the 1% level and (*) at the 5% level. ^a^ Effects coded: negative sum of the below level scale values corresponding to this attribute.

## Data Availability

The data presented in this study are available on request from the corresponding author. The data are not publicly available due to participant confidentiality.

## References

[1] Holland A.M., Haus J.M., Eyler T.B., Duda M.D., Bowman J.L. (2020). Revisiting hunter perceptions toward chronic wasting disease: Changes in behavior over time. Animals.

[2] Harper E.E., Miller C.A., Vaske J.J. (2015). Hunter perceptions of risk, social trust, and management of chronic wasting disease in Illinois. Hum. Dimens. Wildl..

[3] Cooney E.E., Holsman R.H. (2010). Influences on hunter support for deer herd reduction as a chronic wasting disease (CWD) management strategy. Hum. Dimens. Wildl..

[4] Needham M.D., Vaske J.J. (2008). Hunter perceptions of similarity and trust in wildlife agencies and personal risk associated with chronic wasting disease. Soc. Nat. Resour..

[5] Vaske J.J., Miller C.A., Ashbrook A.L., Needham M.D. (2018). Proximity to chronic wasting disease, perceived risk, and social trust in the managing agency. Hum. Dimens. Wildl..

[6] Schroeder S.A., Landon A.C., Cornicelli L., Fulton D.C., McInenly L. (2021). Institutional trust, beliefs, and evaluation of regulations, and management of chronic wasting disease (CWD). Hum. Dimens. Wildl..

[7] Mysterud A., Edmunds D.R. (2019). A review of chronic wasting disease in North America with implications for Europe. Eur. J. Wildl. Res..

[8] Meeks A., Poudyal N.C., Muller L.I., Yoest C. (2022). Hunter acceptability of chronic wasting disease (CWD) management actions in western Tennessee. Hum. Dimens. Wildl..

[9] Vaske J.J. (2010). Lessons learned from human dimensions of chronic wasting disease research. Hum. Dimens. Wildl..

[10] Andersen O., Wam H.K., Mysterud A., Kaltenborn B.P. (2014). Applying typology analyses to management issues: Deer harvest and declining hunter numbers. J. Wildl. Manag..

[11] Ward K.J., Stedman R.C., Luloff A.E., Shortle J.S., Finley J.C. (2008). Categorizing deer hunters by typologies useful to game managers: A latent-class model. Soc. Nat. Resour..

[12] Heberlein T.A. (2004). “Fire in the Sistine Chapel”: How Wisconsin responded to chronic wasting disease. Hum. Dimens. Wildl..

[13] Cornicelli L., Fulton D.C., Grund M.D., Fieberg J. (2011). Hunter perceptions and acceptance of alternative deer management regulations. Wildl. Soc. Bull..

[14] Serenari C., Shaw J., Myers R., Cobb D.T. (2019). Explaining deer hunter preferences for regulatory changes using choice experiments. J. Wildl. Manag..

[15] Wijnen B.F., van der Putten I.M., Groothuis S., de Kinderen R.J., Noben C.Y., Paulus A.T., Hiligsmann M. (2015). Discrete-choice experiments versus rating scale exercises to evaluate the importance of attributes. Expert. Rev. Pharmacoecon. Outcomes Res..

[16] Nylund-Gibson K., Choi A.Y. (2018). Ten frequently asked questions about latent class analysis. Transl. Issues Psychol. Sci..

[17] Spurk D., Hirschi A., Wang M., Valero D., Kauffeld S. (2020). Latent profile analysis: A review and “how to” guide of its application within vocational behavior research. J. Vocat. Behav..

[18] Train K.E. (2002). Discrete Choice Methods with Simulation.

[19] Texas Parks and Wildlife Department Chronic Wasting Disease. https://tpwd.texas.gov/huntwild/wild/diseases/cwd/.

[20] Texas Parks and Wildlife Department New Chronic Wasting Disease Surveillance, Containment Zones Proposed in Five Counties. https://tpwd.texas.gov/newsmedia/releases/?req=20220805a.

[21] Alaimo K., Olson C.M., Frongillo E.A. (1999). Importance of cognitive testing for survey items: An example from food security questionnaires. J. Nutr. Educ..

[22] Heberlein T.A., Stedman R.C. (2009). Socially amplified risk: Attitude and behavior change in response to CWD in Wisconsin deer. Hum. Dimens. Wildl..

[23] Rudolph B.A., Riley S.J. (2014). Factors affecting hunters’ trust and cooperation. Hum. Dimens. Wildl..

[24] Holsman R.H., Petchenik J. (2006). Predicting deer hunter harvest behavior in Wisconsin′s chronic wasting disease eradication zone. Hum. Dimens. Wildl..

[25] Brown T.L., Decker D.J., Major J.T., Curtis P.D., Shanahan J.E., Siemer W.F. (2006). Hunters′ and other citizens′ reactions to discovery of CWD in central New York. Hum. Dimens. Wildl..

[26] Rubino E.C., Pienaar E.F., Soto J.R. (2018). Structuring legal trade in rhino horn to incentivize the participation of South African private landowners. Ecol. Econ..

[27] Dillman D.A., Smyth J.D., Christian L.M. (2014). Internet, Phone, Mail, and Mixed-Mode Surveys: The Tailored Design Method.

[28] Meigs J.B. (2000). Invited commentary: Insulin resistance syndrome? Syndrome X? Multiple metabolic syndrome? A syndrome at all? Factor analysis reveals patterns in the fabric of correlated metabolic risk factors. Am. J. Epidemiol..

[29] Russell J., Grant C.C., Morton S., Denny S., Paine S.J. (2022). Prevalence and predictors of developmental health difficulties within New Zealand preschool-aged children: A latent profile analysis. J. R. Soc. N. Z..

[30] Sforza M., Galbiati A., Zucconi M., Casoni F., Hensley M., Ferini-Strambi L., Castronovo V. (2021). Depressive and stress symptoms in insomnia patients predict group cognitive-behavioral therapy for insomnia long-term effectiveness: A data-driven analysis. J. Affect. Disord..

[31] Siemer W.F., Lauber T.B., Stedman R.C. (2020). New York Hunters’ Perceptions of Chronic Wasting Disease.

[32] Needham M.D., Vaske J.J., Petit J.D. (2017). Risk sensitivity and hunter perceptions of chronic wasting disease risk and other hunting, wildlife, and health risks. Hum. Dimens. Wildl..

[33] Rubino E.C., Serenari C. (2022). Texas stakeholders’ knowledge and perceptions of chronic wasting disease risks: Implications for wildlife agency communications. Hum.-Wildl. Interact..

[34] Jacobson S.K. (2009). Communication Skills for Conservation Professionals.

[35] Stewart C.M., Keller B., Williamson C.R. (2013). Keys to managing a successful archery deer hunt in an urban community: A case study. Hum.-Wildl. Interact..

[36] Rademacher I. (2021). Winning the votes for institutional change: How discursive acts of compromise shaped radical income tax reforms in the United States. Policy Stud..

[37] Adams K.P., Murphy B.P., Ross M.D. (2016). Captive white-tailed deer industry—Current status and growing threat. Wildl. Soc. Bull..

[38] Campbell M., Mackay K.J. (2009). Communicating the role of hunting for wildlife management. Hum. Dimens. Wildl..

